# Our Contemporaries

**Published:** 1916-05

**Authors:** 


### OUR CONTEMPORARIES.

---

We do not usually quote from periodicals which every member of the profession should have, but we take occasion to call attention to the excellence of the Bulletin of the State

Health Dept. and of the Buffalo Sanitary Bulletin. These are mailed free to every physician of the corresponding governmental units, and should be read and studied. The latter, quite aside from its medical interest, sparkles with epigram and wit. For instance: "To CURE is the voice of the past. To PREVENT is the demand of the future" "What effect does an amber necklace have upon goitre? The same effect as a moss agate cuff button." We note that Buffalo last year had a tuberculous mortality of 709, the total deaths being 6,853 (14.83: 1000)—almost exactly 10% of all deaths. Nearly all similar statistics show that tuberculosis is diminishing at almost exactly the same rate as the total mortality. For this reason, some believe that it is not diminishing at all. Speaking of the amber necklace, however, some of the old superstitions really represent accumulated empiric experience, along climatic, therapeutic and other lines. Thus we believe that a reasonable credulity—as to results, not method of operation—is wise, although in this particular, we may say that amber necklaces are not included in our armamentarium.

---

The Indianapolis Medical Journal, March, pays a well deserved tribute to the three Austin Flints, the first two of whom were editors of the Buffalo Medical Journal.

---

Clark Bell, the founder and editor of the Medico-Legal Journal has retired, and is succeeded by Dr. Alfred W. Herzog. The journal will, in the future, appear monthly instead of quarterly.

